# Factors associated with the nutritional status of children less than 5 years of age

**DOI:** 10.1590/S0034-8910.2015049005441

**Published:** 2015-09-29

**Authors:** Teresa Cristina Miglioli, Vania Matos Fonseca, Saint Clair Gomes, Katia Silveira da Silva, Pedro Israel Cabral de Lira, Malaquias Batista

**Affiliations:** I Centro Universitário IBMR. Laureate International Universities. Rio de Janeiro, RJ, Brasil; II Unidade de Pesquisa Clínica. Instituto Fernandes Figueira. Fundação Oswaldo Cruz. Rio de Janeiro, RJ, Brasil; III Departamento de Nutrição. Universidade Federal de Pernambuco. Recife, PE, Brasil; IV Departamento de Pesquisa. Instituto de Medicina Integral Prof. Fernando Figueira. Recife, PE, Brasil

**Keywords:** Vitamin A Deficiency, epidemiology, Anemia, Iron-Deficiency, epidemiology, Body Weight and Measurements, Maternal Nutrition, Maternal and Child Health, Socioeconomic Factors, Health Inequalities, Cross-Sectional Studies

## Abstract

**OBJECTIVE:**

To analyze if the nutritional status of children aged less than five years is related to the biological conditions of their mothers, environmental and socioeconomic factors, and access to health services and social programs.

**METHODS:**

This cross-sectional population-based study analyzed 664 mothers and 790 children using canonical correlation analysis. Dependent variables were characteristics of the children (weight/age, height/age, BMI/age, hemoglobin, and retinol serum levels). Independent variables were those related to the mothers’ nutritional status (BMI, hemoglobin, and retinol serum levels), age, environmental and socioeconomic factors and access to health service and social programs. A < 0.05 significance level was adopted to select the interpreted canonical functions (CF) and ± 0.40 as canonical load value of the analyzed variables.

**RESULTS:**

Three canonical functions were selected, concentrating 89.9% of the variability of the relationship among the groups. In the first canonical function, weight/age (-0.73) and height/age (-0.99) of the children were directly related to the mother’s height (-0.82), prenatal appointments (-0.43), geographical area of the residence (-0.41), and household income *per capita* (-0.42). Inverse relationship between the variables related to the children and people/room (0.44) showed that the larger the number of people/room, the poorer their nutritional status. Rural residents were found to have the worse nutritional conditions. In the second canonical function, the BMI of the mother (-0.48) was related to BMI/age and retinol of the children, indicating that as women gained weight so did their children. Underweight women tended to have children with vitamin A deficiency. In the third canonical function, hemoglobin (-0.72) and retinol serum levels (-0.40) of the children were directly related to the mother’s hemoglobin levels (-0.43).

**CONCLUSIONS:**

Mothers and children were associated concerning anemia, vitamin A deficiency and anthropometric markers. Living in rural areas is a determining factor for the families health status.

## INTRODUCTION

Children and women in their reproductive period are the groups that are most vulnerable to nutritional problems, especially to anemia,^24^ vitamin A deficiency (VAD)^25^ and protein-energy malnutrition (PEM).^2^ Given this, these groups were included as a priority at the United Nations Summit in New York (1990)^a^ and have been targets for actions and family-focused health programs in Brazil and in many countries over the world. However, there are few national or international studies^5^ ^,^ ^10^ ^,^ ^11^ that have investigated the relationship between the nutritional status of the binomial mother and child under the age of five years. In this study, anemia, VAD and anthropometric data are considered proxies for the protein-energy nutritional status.

The health and nutritional situation of the Brazilian population has undergone crucial changes over the last 35 years, which has featured a so-called epidemiological and nutritional transition.^12^ This process of rapid change has transformed the biological, social and geographical distribution of almost all diseases and causes of death. Particularly notable examples of this in the area of nutrition has been the sharp decline of PEM in children and women, and the substantially elevated prevalence of people from these group who are overweight and obese.^12^ ^,^ ^13^

Regarding Brazilian women of reproductive age, the *2006 Pesquisa Nacional de Demografia e Saúde da Criança e da Mulher* (PNDS – National Survey on Demography and Health of Women and Children)^b^ indicated that the prevalence of overweight/obese individuals was already at 59.2%, which is equivalent to the situation of countries that are most affected by this problem, on a global scale.^7^ For children under five years of age, being overweight was observed at 7.3%, which is a value very similar to height-for-age deficits (7.0%).

In the case of anemia and VAD in children up to five years of age, the 2006 PNDS^a^ noted a prevalence of 20.9% and 17.4%, respectively. This research also analyzed women of reproductive age, and found a prevalence of 29.4% for anemia and 12.3% for VAD. The highest rates of anemia, for both children and women, were found in the northeastern region of Brazil.

There are multiple contributing factors for the nutritional status of individuals and populations.^20^ The family and individual’s economic and social environment play a central role in determining their health status. Meager living conditions are typically expressed as low family income, which limits their purchasing power, particularly regarding food, of adverse sanitary conditions, of the geographical area in which they live and of limited and unequal access to health services.^2^

Studying such a topic is relevant because of the interest surrounding its understanding from the point of view of the families, thus providing elements to help create public policies. In Brazil and other countries, field studies usually consider the nutritional status of mothers and children as being isolated groups (women and children), regardless of the family links involved. In order to understand the nutritional status of children, it is necessary to consider them in their family living conditions, which is the bond between the mother, the child and their environment.^16^

The objective of this study was to analyze if the nutritional status of children aged less than five years is related to the biological conditions of their mothers, their environmental and socioeconomic circumstances and their access to health services and social programs.

## METHODS

This is a population-based cross-sectional study. The utilized research database was “*Situação Alimentar Nutricional e de Saúde no Estado de Pernambuco* (Food Nutrition and Health Status in the State of Pernambuco): *Contexto Socioeconômico e de Serviços* (Services and Socioeconomic context) – 3^rd^ Pernambuco State Research on Health and Nutrition” (3^rd^ PESN-PE, 2006).^c^ The data were collected between May 10^th^ and October 25^th^, 2006. Eighteen of the 185 cities in Pernambuco, Northeastern Brazil, were selected, which represents both the urban and rural areas. Families with children under five years of age were considered as the study unit (children-index).

In order to define the sample from the 3^rd^ PESN-PE, 2006, prevalences of malnutrition in children under five years of age (weight-for-age indicator) from the previous study conducted in Pernambuco (2^nd^ PESN-PE, 1997) were used, which were represented by values of 3.2% for urban areas and 6.2% for rural areas, making up a sample of 1,650 children (< 5 years) and 1,909 women of reproductive age.

Based on information from the 3^rd^ PESN-PE database, 2006, children between six and 59 months of age were selected, along with their birth mothers, which formed an *ad-hoc* file that included retinol and hemoglobin data to compose the mother-son paired sample, totaling 664 children and 790 women, which represented 47.9% and 34.8% of the original sample, respectively. Only children who were under the responsibility of their non-pregnant biological mother were included. Observations of the siblings were independently considered, with their inter-correlation being disconsidered. Other details regarding the sampling plan are published in Miglioli et al.^10^ ^,^ ^11^

The hemoglobin dosage was determined using a portable HemoCue photometer (Hemocue, Angelholm, Sweden). Two cut-off points were adopted for classifying anemia: hemoglobin levels below 12 g/dL, for mothers, and under 11 g/dL, for children.^24^

The retinol serum was processed at the *Centro de Investigação em Micronutrientes* (CIMICRON – Micronutrient Research Center) at the Universidade Federal da Paraíba and was analyzed by high performance liquid chromatography. The mothers and children were classified as having VAD when their retinol serum was below 20 μg/dL.^25^

Anthropometric data were measured in duplicate during the interview, while taking into account recommendations given by the World Health Organization (WHO).^23^ Weight-for-age (W/A), height-for-age (H/A) and body mass index (BMI)-for-age (BMI/A) indicators were used for anthropometrically classifying the children, according to the following classification criteria: < -2 z-scores (low W/A, low BMI/A and low H/A); ≥ 2 z-scores (adequate H/A); > 2 z-scores (overweight). For adult women (≥ 20 years), in accordance with criteria from the WHO, the following BMI classification was used:^23^ underweight, for values less than 18.5 kg/m^2^; normal range, for a BMI between 18.5 and 25; and overweight, for a BMI ≥ 25. In adolescent mothers (< 20 years), the BMI/age was used, who were categorized as underweight (< -2 z-scores) and overweight (≥ 1 z-score).^d^

In order to evaluate the observed linear relationship between the variables of mothers and children, the canonical correlation analysis was used. Thistechnique is suitable for studying the interrelationships between sets of multiple dependent and independent variables, and can be used for numeric or categorical data. The purpose of this canonical correlation is to determine a linear combination for each group of variables in a way that maximizes the correlation between the two groups.^8^

The group of dependent variables considered the children’s nutritional status: anthropometric markers (weight-for-age, height-for-age, BMI-for-age), hemoglobin levels and retinol serum. The group of independent variables was formed based on the following characteristics of the mothers: BMI, hemoglobin and retinol levels, age, environmental and socioeconomic factors, and access to health services and social programs. The proposed conceptual model can be seen in the Figure.

**Figure:**
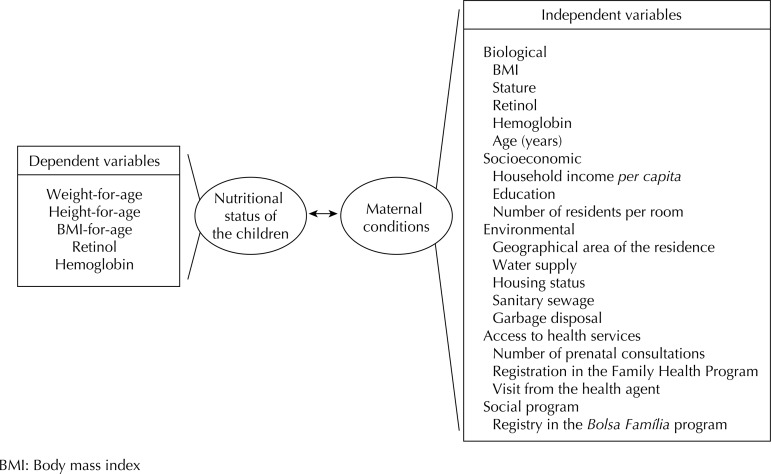
Conceptual model of the canonical correlation analysis between the nutritional status of mothers and their children. Pernambuco, Northeastern Brazil, 2006.

The quality of the model’s fit was evaluated based on the canonical loadings, the Wilks Lambda distribution and a redundancy index. Canonical loadings provided the correlation between the original and canonical variables. The Wilks Lambda distribution jointly identified the significance of the canonical roots. Whereas the redundancy index reported that amount of shared variance could be explained by the canonical functions. The variables that presented a low coefficient of linear relationship were excluded from the model.^8^

In order to select the canonical functions, the criterion for the statistical significance of the function was established at p < 0.05. The canonical load value that defines the variables to be analyzed within each function was established *a priori* at ± 0.40.

The statistical analyses were done using SPSS software (Statistical Package for Social Sciences) version 17.0 (SPSS Inc., Chicago, United States).

This research project was approved by the Committee for Ethics and Research at the Instituto de Medicina Integral Professor Fernando Figueira (IMIP), Process 1,321, in 2004.

## RESULTS

Low levels of hemoglobin were found in 15.0% of mothers and 32.0% of children, with mean values of 13.1 g/dL (SD = 1.3) and 11.4 g/dL (SD = 1.3), respectively. VAD had a 6.9% prevalence in the mothers and 16.1% in the children. The median found in the level of retinol serum (μg/dL) for the mothers was 55.5 (39.6-74.2) and in children it was 37.0 (26.0-51.1).

As regards the anthropometric evaluation of the children, 2.6% were underweight according to the W/A indicator and 1.5% for the BMI/A. 8.9% of the sample were of low height. Instances of overweight individuals was found in 4.7% and 8.6% of the children, according to the W/A and BMI/A indicators, respectively. 4.6% of the mothers were underweight and 44.6% were overweight.

Considering a mean age of 28 years (SD = 6.5) and six years (SD = 3.5) of study, the mothers averagely undertook 6.7 prenatal consultations (SD = 2.2). Based on the collected data, the low household income *per capita* is worth mentioning, the mean of which was 0.3 times the monthly minimum wage (Table 1).

**Table 1:** Biological and sociodemographic characteristics and those related to appointments at health services and social programs undertaken by mothers and their children. Pernambuco, Northeastern Brazil, 2006.

Variable	Mean	SD	n	%	Median	P25;P75
Children (n = 790)						
Weight-for-age (z-score)	0.07	1.1			0.00	-0.65;0.76
Height-for-age (z-score)	-0.50	1.1			-0.49	-1.24;0.27
BMI-for-age (z-score)	0.54	1.1			0.54	-0.17;1.24
Retinol levels (μg/dL)	40.5	18.2			37.0	26.0;51.1
Hemoglobin levels (g/dL)	11.4	1.3			11.5	10.7;12.3
Mothers (n = 664)						
BMI (kg/m^2^)	25.0	4.9			24.4	21.6;27.7
Height (cm)	156.6	6.2			156.6	152.8;160.7
Retinol levels (μg/dL)	57.4	25.7			55.5	39.6;74.2
Hemoglobin levels (g/dL)	13.1	1.3			13.1	12.3;13.8
Age (years)	28.6	6.5			27.9	23.7;32.8
Education (years of study)	6.2	3.5			6.0	4.0;10.0
Number of prenatal consultations	6.7	2.2			7.0	5.0;8.0
Household income *per capita* (minimum wage)	0.3	0.3			0.23	0.11;0.38
Number of residents per room	1.2	0.8			1.0	0.75;1.33
Geographical area of the residence						
Urban			343	51.7		
Rural			321	48.3		
Housing status						
Own home			464	69.9		
Rented house/Other			200	30.1		
Water supply						
General water network			345	52.0		
Garbage disposal						
Public collection			375	56.5		
Registration in the Family Health Program						
Yes			416	62.7		
Registry in the *Bolsa Família* program						
Yes			490	73.8		
Visit from the health agent						
Yes			547	82.5		

BMI: Body mass index

Only half of the households had access to the general water network and public garbage collection. Whereas the majority of the studied population were involved in government social programs, with 62.7% of the families having registered in the Brazilian Family Health Strategy^e^ and 73.8% in the *Bolsa Família* family allowance program^f^ (Table 1).

Table 2 shows the final result from the fitting for selecting the canonical function. The variables presented in the Figure were included for identifying the canonical functions. It was possible to determine five canonical functions (or five pairs of canonical statistical variables), only the first three functions had a significant relationship according to the adopted criterion (p < 0.05), and were therefore selected for the analysis.

**Table 2:** Canonical correlation analysis between the nutritional status of children and biological, sociodemographic conditions, and those related to health services and social programs. Pernambuco, Northeastern Brazil, 2006.

Canonical function	Eigenvalue	Variance %	Variance % cumulative	Canonical Correlation	Wilks lambda	F	p	Redundancy index (%)
1	0.32231	58.8	58.8	0.493	0.61	3.97	< 0.001	24.0
2	0.10578	19.3	78.2	0.309	0.80	2.25	< 0.001	5.0
**3**	**0.06484**	**11.8**	**89.9**	**0.246**	**0.89**	**1.71**	**0.002**	**3.0**
4	0.04072	7.4	97.4	0.197	0.98	1.26	0.16	0.6
5	0.01409	2.6	100.0	0.118	0.99	0.70	0.76	0.2

Limit of the analyzed functions are in bold.

The first canonical function, with a 0.493 correlation, concentrated 58.8% of the variability on the relationship between the groups (mothers and children); the second function represented 19.3%; and the third function, 11.8%. Thus, the sum of the three functions concentrated 89.9% of the observed variability (Table 2).

Table 3 shows the relationships between the dependent variables and the set of independent variables examined by canonical correlation.

**Table 3:** Canonical correlations between variables that are characteristic to children and mothers. Pernambuco, Northeastern Brazil, 2006.

Variable	Canonical function		
	1^st ^load	2^nd^ load	3^rd ^load
Children			
Weight-for-age	-0.73		0.41
Height-for-age	-0.99		
BMI-for-age		-0.62	0.53
Retinol		-0.76	-0.40
Hemoglobin			-0.72
Mothers			
BMI		-0.48	
Height	-0.82		
Hemoglobin			-0.43
Number of prenatal consultations	-0.43		
Geographical area of residence (urban/rural)	-0.41		
Household income *per capita*	-0.42		
Number of residents/rooms	0.44		

BMI: Body mass index

In the first canonical function a strong correlation was observed between weight-for-age (-0.73) and height-for-age (-0.99) of the children with the height of the mother (-0.82), number of prenatal consultations (-0.43), the geographic area of the residence (-0.41) and household income *per capita *(-0.42). The highest canonical loading values, configuring the individual correlation of a variable in a given canonical function, were height/age of children (-0.99) and height of the mother (-0.82). In relation to the geographical area of the residence, those living in the urban areas had the worse nutritional status. However, in that same function, there was an observed inverse relationship between the variables referring to children and number of residents per room (0.44), i.e., the greater the number of people per room, the worse the nutritional status of the mothers and children was, in relation to the analyzed problems.

In the second canonical function, in the group of independent variables, only the mothers’ BMI (-0.48) was related to BMI/age and the level of retinol, indicating an association between a high BMI of the mother and a high BMI of the child. Underweight instances in the mothers were shown to be related to VAD in children.

In the last canonical function, the children’s levels of hemoglobin (-0.72) and retinol (-0.40) were found to be directly related to the mother’s hemoglobin level (-0.43).

## DISCUSSION

The mother’s nutritional status was found to be associated with the family’s environment conditions. The canonical correlation identified an approximate 89.0% association of these factors.

By using this technique, it was possible to investigate the complexity of the interrelationships that exist between nutritional indicators (anthropometrical and biochemical) in the mother-child dyad.

In the set of variables related to nutritional status in the first canonical function, the anthropometric dimension (weight-for-age, height-for-age of children and height of the mother) was the most important, next to the number of pre-natal consultations, geographical area of the residence, household income and number of resident people per room. The second canonical function indicated that, as women’s weight increased, the same happened with their children. Underweight women were also found to have a tendency to bear children with VAD.

The short height of the mother, used as a predictor for nutritional deficit in children, was observed by Silveira et al^16^ (2010) while analyzing 2,075 mothers (18 to 45 years), and their respective children (< 6 years), who were living in the slums of Maceio, AL, Northeastern Brazil. During this study, the children of mothers who were less than 155 cm tall were found to be at twice the risk for height deficit themselves. In a Mexican population of Mayan descent, 70.0% of women were less than 150 cm tall; the children of those mothers were 3.6 times more likely to present height deficit.^19^

Height deficit is an anthropometric characteristic that best represents the epidemiological description of PEM in children and, as it cannot be completely reversed, it becomes a phenotypical manifestation of the problem. Thus, it can be used as a criterion for cartographic mapping of areas characterized by poverty. Usually, its most active pathogenic phase sets in by the age of two years, with it being reflected during the adult life of individuals and populations. Thus, height becomes a historical piece of evidence regarding the epidemiology of a local, regional or national population, going back into the past of the mother and child’s health and the nutrition of their population. Height is determined by both genetic and environmental factors, the deficit of which being a feature that can remain over generations.^20^ Despite being a phenotypic characteristic, it can span between grandparents, parents and adult or growing children, as evidenced by Figueroa et al,^5^ who assessed successive generations in Pernambuco from 1945. This evidence highlights the importance of monitoring malnutrition in women and children, because, besides the immediate consequences of which, malnutrition can affect the nutritional state of future generations.^17^

During a temporal trend study, Menezes et al^9^ analyzed data from three mother and child health surveys in the state of Pernambuco (1991, 1997 and 2006). During these surveys a 65.0% decrease in PEM prevalence (height-for-age) was found in the children, they also verified that a short height (< 150 cm) of the mother meant that the children from whom, less than five years of age, would be three times more likely to present height deficit. Over the period studied, these authors showed that household income *per capita* remained associated with PEM.^9^

Prenatal care, expressed in this study as number of consultations, was strongly associated with the improvement of anthropometric indicators in children, i.e., the higher the number of consultations during pregnancy, the better the conditions of the nutritional status of children are.

Victora et al^21^ highlighted changes in the social determinants of diseases and in the organization of health services over the previous three decades in Brazil. These changes influenced the health indicators of the mother and those of the children’s health and nutrition in a positive way.^21^ They also observed an increase in the percentage of women who underwent more than five prenatal consultations, which went from 40.5% in 1981, to 80.9% in 2006-2007, and decreased from 37.0% in 1974-1975 to 7.0% in 2007 for height deficit among children under five years of age. The regional differences referring to this indicator were equally reduced.

The mean household income *per capita *in this study was 0.31 times the Brazilian monthly minimum salary, which was equivalent to R$105.00/month^g^ in 2006. This result was well below the mean recorded for Brazil (R$396.87) and even for the Northeastern region of the country (R$191.88), according to the PNDS^a^ that was performed in that same year. Selecting the sample based on families with children under five years of age can explain this income gap, whereas families with small children tend to have a smaller income *per capita* than families with no children.^1^ Another explanation would be that families do not declare their full income so as not to exceed the maximum household income rules and remain eligible for the national family benefit system, known as the *Bolsa-Família* program. However, the *Pesquisa Nacional por Amostra de Domicílios (Brazilian National Household Sample Survey – *PNAD/2006) showed that the state of Pernambuco was ranked seventh worst in household income *per capita* in Brazil. In recent decades, despite the national family income *per capita *having increased, regional differences continue to be significant.^h^

The number of resident people per room showed an inverse relationship to the children’s weight-for-age and height-for-age. The mean number of resident people per room was found to be above that observed in Brazil (0.73) in households examined by the 2006 PNDS.^a^ Poor families are generally larger, the resulting larger number of people cohabiting in the same space may signal a potential malnutrition risk. State research, performed in Pernambuco, 1997, showed that households with one or more residents per room were more likely to include children with height deficit.^14^

Living in rural areas was a determining factor for the health status of families, which expressed the persistence of old and profound differences between the contemporary urban-industrial society and the past rural society. The urban-rural dichotomy in Pernambuco and Brazil as a whole has structural processes that have recently become closer.^2^ This phenomenon is also present worldwide.^18^ These regional differences may disappear in Pernambuco in the coming years should this trend continue.

Correlations, such as statistical events, may point to associations between variables; however, these obviously do not explain the processes that link the variables under analysis. Thus, the anthropometric relationship between mothers and children, in addition to the biological component of the genotypes, express conditions between generations who have grown up in different times, in micro- and also macro environments. In these last two dimensions, one generation’s favorable environmental factors can continue into the next generation; in the case of children, this would happen in relation to the nutritional status of micronutrients, such as iron and vitamin A. Due to the fact that feeding children from the ages of two or three is similar to normal adult feeding patterns, the children’s nutritional condition once again is brought closer to that of the mother. In fact, obese people tend to have overweight children, especially when the mother is obese.^3^ ^,^ ^22^

The third canonical function, despite being statistically significant, had a low explanatory power (expressed by the redundancy index). Despite the differences in the methods used in this study, the relationship between the hemoglobin levels of mothers and children has been previously observed in the state of Pernambuco.^10^ ^,^ ^15^ However, Faber et al^5^ found a relative risk of 1.6 for the association of anemia among mothers and children.

Canonical correlation proved to be an interesting technique for analyzing the problem in question. Advantages of this study model were the possibility of adding multiple types of variables in the model, the absence of assumptions regarding the distribution of these variables and the use of functions with more than one dependent variable, which are desirable when studying phenomena in which there is a known inter-relationship between subjects, as is the case in the mother-child relationship. On the other hand, the technique did have its disadvantages, such as: difficulty in interpreting results; lack of statistical tests, even by the flexibility of the assumptions regarding the probability distributions, which support the decision-making process; and differences among the authors who worked with the technique, in relation to thee cut-off points that should be adopted to obtain interpretable canonical functions.

This study shows new multiple regression analysis perspectives (in this case, canonical correlation) of the need-evaluation processes, both for explanatory or independent variables in relation to different outcomes, and between the independent variables with each other. This study more broadly and contextually evaluated the role of exposure factors and outcomes between mothers and their children, which offers a new logic for understanding the nutritional problems in the binomial. As a consensual principle, mothers and children represent recognized segments of biological vulnerability within families, who are in turn distinguished by social inequality that is demarcated by unfavorable conditions, namely in terms of sanitation, housing and even occupational environment.

## Footnotes

a Fundo das Nações Unidas para a Infância. Estratégia para melhorar a nutrição de crianças e mulheres nos países em desenvolvimento. Nova York: Unicef; 1990.

b Ministério da Saúde. Pesquisa Nacional de Demografia e Saúde da Criança e da Mulher PNDS 2006. Brasília (DF); 2008.

c Universidade Federal de Pernambuco. Centro de Ciências da Saúde. Departamento de Nutrição. III Pesquisa Estadual de Saúde e Nutrição: saúde, nutrição, alimentação e condições socioeconômicas e atenção à saúde no estado de Pernambuco. I Inquérito estadual sobre doenças crônicas e agravos não transmissíveis: prevalência e fatores de risco. Recife (PE); 2012.

d Ministério da Saúde. Orientações para a coleta e análise de dados antropométricos em serviços de saúde: norma técnica do Sistema de Vigilância Alimentar e Nutricional – SISVAN. Brasília (DF); 2011.

e Ministério da Saúde, Departamento de Atenção Básica. Atenção básica: saúde da família. Brasília (DF): Ministério da Saúde; 2012 [cited 2015 May 25]. Available from: http://dab.saude.gov.br/portaldab/ape_esf.php

f Ministério da Saúde, Departamento de Atenção Básica. Atenção básica: bolsa família. Brasília (DF): Ministério da Saúde; 2014 [cited 2015 May 30]. Available from: http://bolsafamilia.datasus.gov.br/w3c/bfa.asp

g Equivalent to US$48.81.

h Instituto Brasileiro de Geografia e Estatística. Pesquisa Nacional por Amostra de Domicílios - Pnad 2006. Brasília (DF); 2006 [cited 2014 Oct 15]. Available from: http://www.ibge.gov.br/

## References

[1] Aquilini GH (2014). As mulheres e o trabalho não remunerado na Região Metropolitana de São Paulo. 1^a^ Análise Seade.

[2] Black RE, Allen LH, Bhutta ZA, Caulfield LE, Onis M, Ezzati M (2008). Maternal and child undernutrition: global and regional exposures and health consequences. Lancet.

[3] Castro J (2012). Geografia da fome.

[4] Danielzik S, Czerwinski-Mast M, Langnäse K, Dilba B, Müller MJ (2004). Parental overweight, socioeconomic status and high birth weight are the major determinants of overweight and obesity in 5-7 y-old children: baseline data of the Kiel Obesity Prevention Study (KOPS). Int J Obes Relat Metab Disord.

[5] Faber M, Swanevelder S, Benadé AJ (2005). Is there an association between the nutritional status of the mother and that of her 2-year-old to 5-year-old child?. Int J Food Sci Nutr.

[6] Figueroa JN, Alves JGB, Lira PIC, Batista M (2012). Evolução intergeracional da estatura no Estado de Pernambuco, Brasil, entre 1945 e 2006: 2-aspectos analíticos. Cad Saude Publica.

[7] Finucane MM, Stevens GA, Cowan MJ, Danaei G, Lin JK, Paciorek CJ (2011). National, regional, and global trends in body mass index since 1980: systematic analysis of health examination surveys and epidemiological studies with 960 country-years and 9.1 million participants. Lancet.

[8] Hair JF, Anderson RE, Tathanm RL, Black WC, Sant’Ana AS, Chaves A (2005). Análise multivariada de dados.

[9] Menezes RCE, Lira PIC, Leal VS, Oliveira JS, Santana SCS, Sequeira LAS (2011). Determinantes do déficit estatural em menores de cinco anos no Estado de Pernambuco. Rev Saude Publica.

[10] Miglioli TC, Brito AM, Lira PIC, Figueroa JN, Batista M (2010). Anemia no binômio mãe-filho no Estado de Pernambuco, Brasil. Cad Saude Publica.

[11] Miglioli TC, Fonseca VM, Gomes S, Lira PIC, Batista M (2013). Deficiência de vitamina A em mães e filhos no estado de Pernambuco. Cienc Saude Coletiva.

[12] Mondini L, Gimeno SGA, Taddei JA, Lang RMF, Longo-Silva G, Toloni MHA (2011). Transição nutricional: significado, determinantes e prognóstico. Nutrição em saúde pública.

[13] Monteiro CA, Benicio MH, Konno SC, Silva AC, Lima AL, Conde WL (2009). Causas para o declínio da desnutrição infantil no Brasil, 1996-2007. Rev Saude Publica.

[14] Rissin A, Figueroa JN, Benício MHD A, Batista M (2011). Retardo estatural em menores de cinco anos: um estudo “baseline”. Cienc Saude Coletiva.

[15] Silva SCL, Batista M, Miglioli TC (2008). Prevalência e fatores de risco de anemia em mães e filhos no Estado de Pernambuco. Rev Bras Epidemiol.

[16] Silveira KBR, Alves JFR, Ferreira HS, Sawaya AL, Florêncio TMMT (2010). Associação entre desnutrição em crianças moradoras de favelas, estado nutricional materno e fatores socioambientais. J Pediatr (Rio J).

[17] Souganidis ES (2011). The importance of early nutritional intervention. How maternal and child undernutrition in early life can affect health in later years. Sight Life.

[18] Van de Poel E, O’Donnell O, Van Doorslaer E (2007). Are urban children really healthier? Evidence from 47 developing countries. Soc Sci Med.

[19] Varela-Silva MI, Azcorra H, Dickinson F, Bogin B, Frisancho AR (2009). Inﬂuence of maternal stature, pregnancy age, and infant birth weight on growth during childhood in Yucatan, Mexico: a test of the intergenerational effects hypothesis. Am J Human Biol.

[20] Victora CG, Adair L, Fall C, Hallal PC, Martorell R, Richter L, Sachdev HS (2008). Maternal and child undernutrition: consequences for adult health and human capital. Lancet.

[21] Victora CG, Aquino EML, Leal MC, Monteiro CA, Barros FC, Szwarcwald CL (2011). Saúde de mães e crianças no Brasil: progressos e desafios. Lancet.

[22] Williams SM, Taylor RW, Taylor. BJ (2013). Secular changes in BMI and the associations between risk factors and BMI in children born 29 years apart. Pediatr Obes.

[23] World Health Organization (1995). Physical Status: The use and interpretation of anthropometry.

[24] World Health Organization (2008). Worldwide prevalence of anaemia 1993-2005: WHO global database on anaemia.

[25] World Health Organization (2009). Global prevalence of vitamin A deficiency in populations at risk 1995-2005. WHO Global database on vitamin A deficiency.

