# Supplementary material for: Factors associated with the nutritional status of children less than 5 years of age
Source: Rev Saude Publica. 2015 Sep 29;49:59. doi: 10.1590/S0034-8910.2015049005441 (PMC4617433; doi:10.1590/S0034-8910.2015049005441)
Supplement: Translation

# Translation: Fatores associados ao estado nutricional de crianças menores de cinco anos

**Authors:** Teresa Cristina Miglioli, Vania Matos Fonseca, Saint Clair Gomes, Katia Silveira da Silva, Pedro Israel Cabral de Lira, Malaquias Batista

**Affiliations:** I Centro Universitário IBMR. Laureate International Universities. Rio de Janeiro, RJ, Brasil; I I Unidade de Pesquisa Clínica. Instituto Fernandes Figueira. Fundação Oswaldo Cruz. Rio de Janeiro, RJ, Brasil; II I Departamento de Nutrição. Universidade Federal de Pernambuco. Recife, PE, Brasil; IV Departamento de Pesquisa. Instituto de Medicina Integral Prof. Fernando Figueira. Recife, PE, Brasil

**Keywords:** Deficiência de Vitamina A, epidemiologia, Anemia Ferropriva, epidemiologia, Pesos e Medidas Corporais, Nutrição Materna, Saúde Materno-Infantil, Fatores Socioeconômicos, Desigualdades em Saúde, Estudos Transversais

## Abstract

**OBJETIVO:**

Analisar se o estado nutricional de crianças menores de cinco anos está relacionado a condições biológicas de suas mães, a fatores ambientais e socioeconômicos e ao acesso a serviços de saúde e programas sociais.

**MÉTODOS:**

Este estudo transversal, de base populacional, analisou 664 mães e 790 filhos, utilizando análise de correlação canônica, tendo como variáveis dependentes as características dos filhos (peso/idade, estatura/idade, índice de massa corporal/idade, níveis de hemoglobina e retinol sérico). Como variáveis independentes, as relacionadas ao estado nutricional das mães (índice de massa corporal, níveis de hemoglobina e retinol sérico), idade materna, fatores ambientais, socioeconômicos, acesso a serviços de saúde e programas sociais. Adotou-se nível de significância < 0,05 para seleção das funções canônicas interpretadas e ± 0,40 como valor de carga canônica das variáveis analisadas.

**RESULTADOS:**

Foram selecionadas três funções canônicas, concentrando 89,9% da variabilidade da relação entre os grupos. Na primeira, peso/idade (-0,73) e estatura/idade (-0,99) dos filhos relacionaram-se diretamente com estatura materna (-0,82), consultas pré-natais (-0,43), área geográfica de moradia (-0,41) e renda familiar *per capita* (-0,42). A relação inversa entre variáveis referentes aos filhos e moradores/cômodo (0,44) mostrou que, quanto maior o número de pessoas/cômodo, mais deficiente o estado nutricional. Residentes na área rural apresentaram piores situações nutricionais. Na segunda função, índice de massa corporal materno (-0,48) relacionou-se com índice de massa corporal/idade e retinol dos filhos, indicando que, à medida que as mulheres aumentavam de peso, o mesmo acontecia com seus filhos e que mulheres de baixo peso tendiam a ter filhos com deficiência de vitamina A. Na terceira função, os níveis de hemoglobina (-0,72) e retinol (-0,40) dos filhos estiveram diretamente relacionados ao nível de hemoglobina materna (-0,43).

**CONCLUSÕES:**

Mães e filhos estavam associados em relação à anemia, deficiência de vitamina A e marcadores antropométricos. Residir na área rural ainda é um discriminante na situação de saúde das famílias.

## INTRODUÇÃO

Mulheres no período reprodutivo e crianças são os grupos mais vulneráveis aos problemas nutricionais, sobretudo para anemia,^24^ deficiência de vitamina A (DVA)^25^ e desnutrição energético-protéica (DEP).^2^ Diante disso, esses grupos foram incluídos como prioridade na Reunião de Cúpula das Nações Unidas em Nova York (1990)^a^ e têm sido alvos de ações e programas de saúde com enfoque familiar no Brasil e em vários países do mundo. Porém, poucos estudos nacionais e internacionais^5^ ^,^ ^10^ ^,^ ^11^ investigam a relação entre o estado nutricional do binômio mãe e filho menor de cinco anos. No presente estudo, anemia, DVA e dados antropométricos são considerados *proxi* do estado de nutrição energético-protéico.

No Brasil, a situação de saúde e nutrição da população apresentou mudanças cruciais nos últimos 35 anos, caracterizando a chamada transição epidemiológica e nutricional.^12^ Esse processo de rápidas mudanças transformou a distribuição geográfica, social e biológica de quase todas as doenças e causas de morte, sendo particularmente notáveis, na área de nutrição, o acentuado declínio da DEP em crianças e mulheres e a substancial elevação das prevalências de sobrepeso e obesidade nesses mesmos grupos.^12^ ^,^ ^13^

No caso das mulheres brasileiras em idade reprodutiva, a Pesquisa Nacional de Demografia e Saúde da Criança e da Mulher (PNDS) de 2006^b^ indicou que a prevalência do sobrepeso/obesidade já se encontrava em 59,2%, equivalente à situação dos países mais afetados pelo problema, em escala mundial.^7^ Para os menores de cinco anos, excesso de peso foi observado em 7,3%, valores bem semelhantes aos *déficits* de altura-para-idade (7,0%).

Quanto à anemia e DVA em crianças até cinco anos, a PNDS 2006^a^ apontou prevalências de 20,9% e 17,4%, respectivamente. A pesquisa analisou também mulheres em idade reprodutiva, encontrando prevalências de 29,4% para anemia e 12,3% para DVA. As maiores taxas de anemia, tanto para as crianças como para as mulheres, foram encontradas na região Nordeste.

A situação nutricional de indivíduos e populações é multicausal.^20^ O ambiente social e econômico do indivíduo e de sua família desempenha papel central na determinação da situação de saúde. Condições de vida precárias normalmente se expressam na baixa renda familiar, no limitado poder de compra, particularmente dos alimentos, nas condições adversas de saneamento, na área geográfica de moradia e no limitado e desigual acesso a serviços de saúde.^2^

O estudo do agregado é relevante pelo interesse em compreendê-lo a partir do enfoque familiar, fornecendo elementos para formulação de políticas públicas. Habitualmente, no Brasil e em outros países, estudos de campo consideram o estado de nutrição de mães e filhos como grupos isolados (mulheres e crianças), independentemente de vínculos familiares. Para a compreensão da situação nutricional das crianças, é necessário considerá-las em seu *habitat* familiar, sendo a mãe o elo entre a criança e o ambiente.^16^

O objetivo deste trabalho foi analisar se o estado nutricional de crianças menores de cinco anos está relacionado a condições biológicas de suas mães, a fatores ambientais e socioeconômicos e ao acesso a serviços de saúde e programas sociais.

## MÉTODOS

Trata-se de estudo transversal, de base populacional. Foi utilizado o banco de dados da pesquisa “Situação Alimentar Nutricional e de Saúde no Estado de Pernambuco: Contexto Socioeconômico e de Serviços – III Pesquisa Estadual de Saúde e Nutrição” (III PESN-PE 2006).^c^ Os dados foram coletados entre 10 de maio e 25 de outubro de 2006. Dos 185 municípios de Pernambuco, foram selecionados 18, representando as áreas urbanas e rurais. Considerou-se como unidade de estudo a família com menores de cinco anos (crianças-índice).

Para a definição da amostra da III PESN-PE 2006, foram utilizadas prevalências de desnutrição em menores de cinco anos (indicador peso-para-idade) do estudo anterior realizado em Pernambuco (II PESN-PE 1997), representadas por valores de 3,2% para área urbana e 6,2% para a área rural, perfazendo uma amostra de 1.650 crianças (< 5 anos) e 1.909 mulheres em idade reprodutiva.

A partir do banco da III PESN-PE 2006, foram selecionadas crianças entre seis e 59 meses de idade, conjuntamente com suas mães biológicas, formando um arquivo *ad-hoc*, incluindo dados de retinol e hemoglobina, de modo a compor o pareamento amostral mãe-filho, totalizando 790 crianças e 664 mulheres, que representam, respectivamente, 47,9% e 34,8% da amostra original. Foram incluídas somente crianças que estavam sob a responsabilidade de sua mãe biológica não grávida. Os irmãos foram considerados observações independentes, desconsiderando sua inter-correlação. Outros detalhes sobre o plano amostral encontram-se publicados em Miglioli et al.^10^ ^,^ ^11^

A dosagem de hemoglobina foi realizada em fotômetro portátil HemoCue (Hemocue, Angelholm, Suécia). Dois pontos de corte foram adotados para classificar a anemia: nível de hemoglobina abaixo de 12 g/dL, para mães, e abaixo de 11 g/dL, para filhos.^24^

O retinol sérico foi processado no Centro de Investigação em Micronutrientes (CIMICRON) da Universidade Federal da Paraíba e analisado por cromatografia líquida de alta performance. Mães e filhos foram classificados com DVA quando apresentavam concentração sérica de retinol abaixo de 20 mg/dL.^25^

Os dados antropométricos foram aferidos em duplicata no momento da entrevista, atendendo às recomendações da Organização Mundial da Saúde (OMS).^23^ Para classificação antropométrica das crianças foram utilizados os indicadores peso-para-idade (P/I), estatura-para-idade (E/I) e índice de massa corporal (IMC)-para-idade (IMC/I), obedecendo aos seguintes critérios de classificação: < -2 escores z (baixo P/I, baixo IMC/I e baixa E/I); ≥ 2 escores z (E/I adequada); > 2 escores z (excesso de peso). Para as mulheres adultas (≥ 20 anos), foi utilizada a seguinte classificação de IMC, de acordo com os critérios da OMS:^23^ baixo peso, para valores inferiores a 18,5 kg/m^2^; eutrofia, para IMC entre 18,5 e 25; e excesso de peso, para IMC ≥ 25. Nas mães adolescentes (< 20 anos), utilizou-se o IMC/Idade, categorizado como baixo peso (< -2 escores z) e excesso de peso (≥ 1 escore-z).^d^

Para avaliar a relação entre o conjunto de variáveis observadas de mães e filhos, utilizou-se a análise de correlação canônica. Esta técnica é adequada para o estudo de inter-relações entre conjuntos de múltiplas variáveis dependentes e independentes, podendo ser utilizada para dados numéricos ou categóricos. O objetivo da correlação canônica é determinar uma combinação linear para cada grupo de variáveis, de forma que maximize a correlação entre os dois grupos.^8^

O grupo de variáveis dependentes considerou o estado nutricional dos filhos: marcadores antropométricos (peso-para-idade, estatura-para-idade, IMC-para-idade), níveis de hemoglobina e retinol sérico. O grupo de variáveis independentes foi formado pelas seguintes características das mães: IMC, níveis de hemoglobina e retinol, idade, fatores ambientais, socioeconômicos, acesso a serviços de saúde e programas sociais. O modelo conceitual proposto encontra-se na Figura.

**Figura:** Modelo conceitual da análise de correlação canônica entre estado nutricional de mães e filhos. Pernambuco, 2006.

A qualidade do ajuste do modelo foi avaliada a partir das cargas canônicas, Lambda de Wilks e índice de redundância. As cargas canônicas forneceram a correlação entre as variáveis originais e as variáveis canônicas. O Lambda de Wilks identificou a significância das raízes canônicas conjuntamente. Já o índice de redundância, informou a quantidade de variância compartilhada que pode ser explicada pelas funções canônicas. As variáveis que apresentaram baixo coeficiente de relação linear foram retiradas do modelo.^8^

Para seleção das funções canônicas, foi estabelecido o critério de significância estatística da função em p < 0,05. O valor de carga canônica que define as variáveis a serem analisadas dentro de cada função foi estabelecido *a priori* como ± 0,40.

As análises estatísticas foram feitas no programa SPSS (*Statistical Package for Social Sciences*) versão 17.0 (SPSS Inc., Chicago, Estados Unidos).

O projeto de pesquisa foi aprovado pelo Comitê de Ética e Pesquisa do Instituto de Medicina Integral Prof. Fernando Figueira (IMIP), Processo 1.321 de 2004.

## RESULTADOS

Níveis baixos de hemoglobina foram encontrados em 15,0% das mães e 32,0% das crianças, com valores médios de 13,1 g/dL (DP = 1,3) e 11,4 g/dL (DP = 1,3), respectivamente. A DVA atingiu 6,9% das mães e 16,1% das crianças. A mediana encontrada no nível de retinol sérico (mg/dL) das mães foi 55,5 (39,6-74,2) e nas crianças 37,0 (26,0-51,1).

Em relação à avaliação antropométrica das crianças, 2,6% estavam abaixo do peso pelo indicador P/I e 1,5% pelo IMC/I. Baixa estatura atingiu 8,9% da amostra. Excesso de peso foi encontrado em 4,7% e 8,6% das crianças, pelos indicadores P/I e IMC/I, respectivamente. Quanto às mães, 4,6% estavam abaixo do peso e 44,6% com peso elevado.

Com média de idade de 28 anos (DP = 6,5) e seis anos (DP = 3,5) de estudo, as mães realizaram, em média, 6,7 consultas pré-natais (DP = 2,2). Destaca-se, no conjunto de informações, a baixa renda familiar *per capita*, cuja média era de 0,3 salário mínimo (Tabela 1).

**Tabela 1:** Características biológicas, sociodemográficas e relativas aos atendimentos nos serviços de saúde e programas sociais das mães e filhos. Pernambuco, 2006.

Variável	Média	DP	n	%	Mediana	P25;P75
Filhos (n = 790)						
Peso-para-idade (escore z)	0,07	1,1			0,00	-0,65;0,76
Estatura-para-idade (escore z)	-0,50	1,1			-0,49	-1,24;0,27
IMC-para-idade (escore z)	0,54	1,1			0,54	-0,17;1,24
Níveis de retinol (mg/dL)	40,5	18,2			37,0	26,0;51,1
Níveis de hemoglobina (g/dL)	11,4	1,3			11,5	10,7;12,3
Mães (n = 664)						
IMC (kg/m^2^)	25,0	4,9			24,4	21,6;27,7
Estatura (cm)	156,6	6,2			156,5	152,8;160,7
Níveis de retinol (mg/dL)	57,4	25,7			55,5	39,6;74,2
Níveis de hemoglobina (g/dL)	13,1	1,3			13,1	12,3;13,8
Idade (anos)	28,6	6,5			27,9	23,7;32,8
Escolaridade (anos de estudo)	6,2	3,5			6,0	4,0;10,0
Quantidade de consultas no pré-natal	6,7	2,2			7,0	5,0;8,0
Renda familiar *per capita* (salário mínimo)	0,3	0,3			0,23	0,11;0,38
Quantidade de moradores por cômodos	1,2	0,8			1,0	0,75;1,33
Área geográfica de moradia						
Urbano			343	51,7		
Rural			321	48,3		
Regime de moradia						
Casa própria			464	69,9		
Casa alugada/Outros			200	30,1		
Abastecimento de água						
Com rede geral			345	52,0		
Destino do lixo						
Com coleta pública			375	56,5		
Cadastro no Programa Saúde da Família						
Sim			416	62,7		
Inscrição no Programa Bolsa Família						
Sim			490	73,8		
Visita do agente de saúde						
Sim			547	82,5		

IMC: Índice de massa corporal

Apenas metade dos domicílios tinha acesso à rede geral de água e coleta pública de lixo. Já os programas sociais do governo atendem a maior parte da população estudada, com 62,7% das famílias cadastradas na Estratégia Saúde da Família^e^ e 73,8% inscritas no Programa Bolsa Família^f^ (Tabela 1).

A Tabela 2 apresenta o resultado final do ajuste para seleção da função canônica. As variáveis apresentadas na Figura foram incluídas para a identificação das funções canônicas. Foi possível determinar cinco funções canônicas (ou cinco pares de variáveis estatísticas canônicas) e apenas as três primeiras funções tiveram relação significativa segundo o critério adotado (p < 0,05), sendo, portanto, as selecionadas para a análise.

**Tabela 2:** Análise da correlação canônica entre o estado nutricional de filhos e condições biológicas, sociodemográficas, relativas aos serviços de saúde e programas sociais das mães. Pernambuco, 2006.

Função canônica	Autovalor	% Variância	% Variância cumulativa	Correlação Canônica	Lambda de Wilks	F	p	Índice de redundância (%)
1	0,32231	58,8	58,8	0,493	0,61	3,97	< 0,001	24,0
2	0,10578	19,3	78,2	0,309	0,80	2,25	< 0,001	5,0
**3**	**0,06484**	**11,8**	**89,9**	**0,246**	**0,89**	**1,71**	**0,002**	**3,0**
4	0,04072	7,4	97,4	0,197	0,98	1,26	0,16	0,6
5	0,01409	2,6	100,0	0,118	0,99	0,70	0,76	0,2

Limite das funções analisadas apresentado em negrito.

A primeira função canônica, com correlação de 0,493, concentrou 58,8% da variabilidade da relação entre os grupos (mães e filhos); a segunda função representou 19,3%; e a terceira função, 11,8%. Assim, a soma das três funções concentrou 89,9% da variabilidade observada (Tabela 2).

A Tabela 3 mostra as relações entre o conjunto de variáveis dependentes e o conjunto das independentes examinadas pela correlação canônica.

**Tabela 3:** Correlações canônicas entre variáveis características aos filhos e mães. Pernambuco, 2006.

Variável	Função canônica		
	1ª carga	2ª carga	3ª carga
Filhos			
Peso-para-idade	-0,73		0,41
Estatura-para-idade	-0,99		
IMC-para-idade		-0,62	0,53
Retinol		-0,76	-0,40
Hemoglobina			-0,72
Mães			
IMC		-0,48	
Estatura	-0,82		
Hemoglobina			-0,43
Quantidade de consultas no pré-natal	-0,43		
Área geográfica de moradia (rural/urbana)	-0,41		
Renda familiar* per capita*	-0,42		
Quantidade de moradores/cômodos	0,44		

IMC: Índice de massa corporal

Na primeira função canônica foi observada forte correlação entre peso-para-idade (-0,73) e estatura-para-idade (-0,99) dos filhos com a estatura da mãe (-0,82), número de consultas pré-natais (-0,43), área geográfica de moradia (-0,41) e renda familiar* per capita *(-0,42). Os maiores valores de cargas canônicas, configurando a correlação individual de uma variável em determinada função canônica, foram a estatura/idade dos filhos (-0,99) e estatura materna (-0,82). Em relação à área geográfica de moradia, residentes na área rural encontravam-se em piores situações nutricionais. Entretanto, nessa mesma função, foi observada relação inversa entre as variáveis referentes aos filhos e número de moradores por cômodo (0,44), ou seja, quanto maior o número de pessoas por cômodo, pior o estado nutricional de mães e filhos, em relação aos problemas analisados.

Na segunda função canônica, no grupo das variáveis independentes, apenas o IMC das mães (-0,48) relacionou-se com o IMC/idade e o nível de retinol dos filhos, indicando que maior IMC materno estava associado a maior IMC da criança. Baixo peso materno mostrou-se relacionado à DVA em crianças.

Na última função canônica interpretada, o nível de hemoglobina (-0,72) e de retinol (-0,40) dos filhos encontrava-se diretamente relacionado ao nível de hemoglobina materno (-0,43).

## DISCUSSÃO

O estado nutricional materno associou-se às condições do ambiente familiar. A correlação canônica identificou associação de aproximadamente 89,0% desses fatores.

Utilizando-se essa técnica, foi possível investigar a complexidade das inter-relações existentes entre indicadores nutricionais (antropométricos e bioquímicos) na díade mãe-filho.

No conjunto das variáveis referentes ao estado nutricional, na primeira função canônica, a dimensão antropométrica (peso-para-idade, estatura-para-idade dos filhos e estatura materna) foi a mais importante, ao lado do número de consultas pré-natais, área geográfica de moradia, renda familiar e número de pessoas por cômodos. A segunda função canônica indicou que, à medida que as mulheres aumentavam de peso, o mesmo acontecia com seus filhos. Verificou-se ainda que mulheres de baixo peso tendiam a ter filhos com DVA.

A baixa estatura materna como preditor do *déficit *nutricional em crianças foi observada por Silveira et al^16^ (2010) ao analisarem 2.075 mães (18 a 45 anos) e respectivos filhos (< 6 anos) residentes em favelas de Maceió, AL, encontrando nas mães com estatura inferior a 155 cm, risco duas vezes maior para a ocorrência de *déficit* estatural nos filhos. Em população mexicana de descendência Maia, 70,0% das mulheres apresentavam estatura abaixo de 150 cm e os filhos dessas mães tiveram 3,6 vezes a probabilidade de apresentarem *déficit* estatural.^19^

O *déficit* estatural é a característica antropométrica que melhor representa o quadro epidemiológico da DEP em crianças e, como não pode ser completamente revertido, torna-se uma manifestação fenotípica do problema. Assim, pode ser usado como critério de mapeamento cartográfico de ambientes marcados pela pobreza. Habitualmente, sua fase patógena mais ativa se instala até os dois anos de idade, refletindo-se na vida adulta de indivíduos e populações. Assim, a altura torna-se um testemunho histórico da epidemiologia de uma comunidade local, regional ou nacional, retrocedendo ao passado da saúde materno-infantil e nutrição de sua população. A altura é determinada tanto por fatores genéticos quanto ambientais e o *déficit* estatural é uma sequela que pode se manter por gerações.^20^ Mesmo sendo uma característica fenotípica, pode se prolongar entre avós, pais e filhos adultos ou em crescimento, conforme mostrado por Figueroa et al,^5^ que avaliou gerações sucessivas em Pernambuco a partir de 1945. Essas evidências destacam a importância do monitoramento da desnutrição em mulheres e crianças, pois, além de suas consequências imediatas, a desnutrição pode influenciar o estado nutricional de gerações futuras.^17^

Em estudo de tendência temporal, Menezes et al,^9^ analisaram dados de três pesquisas estaduais de saúde materno-infantil em Pernambuco (1991, 1997 e 2006), e encontraram diminuição de 65,0% na prevalência da DEP (altura-para-idade) em crianças, verificando-se que a baixa estatura materna (< 150 cm) representou chance três vezes maior de *déﬁcit* estatural nos filhos menores de cinco anos. Esses autores mostraram ainda que, ao longo do período estudado, a renda familiar *per capita* manteve-se associada à DEP.^9^

A assistência pré-natal, aqui expressa pelo número de consultas realizadas, foi fortemente associada à melhoria dos indicadores antropométricos dos filhos, ou seja, quanto maior o número de consultas na gestação, melhores as condições do estado nutricional dos filhos.

Victora et al^21^ apontaram mudanças nos determinantes sociais das doenças e na organização dos serviços de saúde nas últimas três décadas no Brasil. Essas mudanças afetaram positivamente os indicadores de saúde materna e de saúde e nutrição infantil.^21^ Também observaram aumento no percentual de mulheres que realizaram mais de cinco consultas no pré-natal, que passou de 40,5%, em 1981, para 80,9%, em 2006-2007, e diminuição de 37,0% em 1974-1975 para 7,0% em 2007 do *déficit estatural* entre crianças menores de cinco anos. As diferenças regionais referentes a esse indicador foram igualmente reduzidas.

A média da renda familiar *per capita* no presente estudo foi de 0,31 salário mínimos, equivalente à R$105,00/mês^g^ no ano de 2006. Era um resultado bem inferior às médias verificadas para o Brasil (R$396,87) e até mesmo para o Nordeste (R$191,88), segundo a PNDS^a^ realizada no mesmo ano. A seleção da amostra baseada em famílias com crianças menores de cinco anos pode justificar essa diferença de renda, visto que famílias com filhos pequenos tendem a ter uma renda *per capita* menor do que aquelas sem filhos.^1^ Outra justificativa seria uma subdeclaração de renda para não exceder as regras de renda familiar mínima para inclusão no Programa Bolsa-Família. Ainda, a Pesquisa Nacional por Amostra de Domicílios *(*Pnad/2006) mostrou que Pernambuco ocupava a sétima pior posição do rendimento familiar *per capita* do País. Apesar de, nas últimas décadas, o rendimento familiar *per capita* nacional ter apresentado elevação, as diferenças regionais ainda continuam marcantes.^h^

O número de pessoas por cômodo apresentou relação inversa ao peso-para-idade e estatura-para-idade dos filhos. A média encontrada de moradores por cômodo foi acima da observada no País (0,73) nos domicílios analisados pela PNDS 2006.^a^ Famílias pobres geralmente são mais numerosas e o número de pessoas que coabitam o mesmo espaço pode ser um potencial sinalizador de risco para a desnutrição. Pesquisa estadual realizada em Pernambuco em 1997 mostrou que os domicílios com um ou mais moradores por cômodo apresentaram maiores chances de terem crianças com *déficit* estatural.^14^

Residir na área rural foi um discriminante na situação de saúde das famílias, expressando a persistência de antigas e profundas diferenciações entre a sociedade urbano-industrial contemporânea e a sociedade rural do passado. A dicotomia urbano-rural em Pernambuco e no Brasil como um todo caracteriza processos estruturais que recentemente estão sendo aproximados.^2^ Esse fenômeno também ocorre mundialmente.^18^ As diferenças regionais poderão desaparecer em Pernambuco nos próximos anos, caso essa tendência seja mantida.

As correlações, como eventos estatísticos, podem apontar associações entre variáveis, mas, evidentemente, não explicam os processos que vinculam as variáveis sob análise. Assim, as relações antropométricas entre mães e filhos, além do componente biológico dos genótipos, expressam as condições entre gerações que cresceram em épocas distintas, em micro e macroambientes também distintos. Nestas duas últimas dimensões, os fatores ambientais favoráveis de uma geração podem se manter na geração seguinte; no caso dos filhos, isso aconteceria em relação ao estado nutricional dos micronutrientes, como ferro e vitamina A. Como a alimentação da criança a partir de dois ou três anos assemelha-se à alimentação usual do adulto, ocorre novamente aproximação entre a condição nutricional da criança e da mãe. De fato, pessoas obesas tendem a ter filhos com excesso de peso, sobretudo quando a obesidade é materna.^3^ ^,^ ^22^

A terceira função canônica, embora estatisticamente significativa, apresentou baixo poder explicativo (expresso pelo índice de redundância). Apesar das diferenças nos métodos empregados neste estudo, a relação entre os níveis de hemoglobina das mães e dos filhos foi previamente observada no Estado de Pernambuco.^10^ ^,^ ^15^ Ainda, Faber et al^5^ encontraram risco relativo de 1,6 para a associação da anemia entre mães e filhos.

A correlação canônica apresentou-se como uma técnica interessante para a análise do problema em questão. A possibilidade de inclusão de múltiplos tipos de variáveis no modelo, a ausência de pressupostos quanto à distribuição dessas variáveis e a formação de funções com mais de uma variável dependente, caracterizam como vantagens quando se pretende estudar fenômenos em que sabidamente existe inter-relação entre sujeitos, como é o caso da relação mãe-filho. Por outro lado, percebem-se como desvantagens da técnica: dificuldade para interpretação dos resultados; carência de testes estatísticos, até mesmo pela própria flexibilidade dos pressupostos quanto às distribuições de probabilidade, que apoiassem o processo de decisão; e divergência entre os autores que trabalham com a técnica quanto aos pontos de corte que devem ser adotados para obtenção de funções canônicas interpretáveis.

Este estudo mostra novas perspectivas de análise de regressão múltipla (no caso, correlação canônica) dos processos carenciais, tanto no que se refere às variáveis explanatórias ou independentes em relação aos diferentes desfechos, quanto entre as variáveis independentes entre si. De forma mais ampla e contextualizada, o presente estudo avalia o papel dos fatores de exposição e desfechos entre as mães e seus filhos, acrescentando uma nova lógica para a compreensão dos problemas nutricionais no binômio. Como princípio consensual, mães e filhos representam segmentos de reconhecida vulnerabilidade biológica, dentro de famílias que, por sua vez, distinguem-se pela desigualdade social demarcada pelas condições desfavoráveis em termos de saneamento, de habitação e até de ambiente ocupacional.

## Footnotes

a Fundo das Nações Unidas para a Infância. Estratégia para melhorar a nutrição de crianças e mulheres nos países em desenvolvimento. Nova York: Unicef; 1990.

b Ministério da Saúde. Pesquisa Nacional de Demografia e Saúde da Criança e da Mulher PNDS 2006. Brasília (DF); 2008.

c Universidade Federal de Pernambuco. Centro de Ciências da Saúde. Departamento de Nutrição. III Pesquisa Estadual de Saúde e Nutrição: saúde, nutrição, alimentação e condições socioeconômicas e atenção à saúde no estado de Pernambuco. I Inquérito estadual sobre doenças crônicas e agravos não transmissíveis: prevalência e fatores de risco. Recife (PE); 2012.

d Ministério da Saúde. Orientações para a coleta e análise de dados antropométricos em serviços de saúde: norma técnica do Sistema de Vigilância Alimentar e Nutricional – SISVAN. Brasília (DF); 2011.

e Ministério da Saúde, Departamento de Atenção Básica. Atenção básica: saúde da família. Brasília (DF): Ministério da Saúde; 2012 [citado 2015 mai 25]. Disponível em: http://dab.saude.gov.br/portaldab/ape_esf.php

f Ministério da Saúde, Departamento de Atenção Básica. Atenção básica: bolsa família. Brasília (DF): Ministério da Saúde; 2014 [citado 2015 mai 30].Disponível em: http://bolsafamilia.datasus.gov.br/w3c/bfa.asp

g Equivalente a US$48,81.

h Instituto Brasileiro de Geografia e Estatística. Pesquisa Nacional por Amostra de Domicílios - Pnad 2006. Brasília (DF); 2006 [citado 2014 out 15]. Disponível em: http://www.ibge.gov.br/
